# Comparison of predictors of survival among fulminant myocarditis patients undergoing veno-arterial extracorporeal membrane oxygenation in the adult and pediatric populations

**DOI:** 10.1097/MS9.0000000000002636

**Published:** 2024-10-16

**Authors:** Yomna E. Dean, Mohamed Doma, Ahson Afzal, Sameh Samir Elawady, Rafeek W. Elmezayen, Bdoor Ahmed A. Bamousa, Naila Iqbal, Muluken Zeleke Megiso, Sriharsha Kodurum, Adham Ramadan, Mahmoud El Bahaie, Ahmed Magdi, Fatima Afzal, Helmy Badr, Basant Katamesh, Dina Ismail, Yasser Etman, Yusef Hazimeh, Edward Darling, Hani Aiash

**Affiliations:** a Faculty of Medicine, Alexandria University, Egypt; b Dow Medical College, Karachi, Pakistan; c Neuro-Endovascular Surgery, Medical University of South Carolina (MUSC), SC, USA; d Kafr El-sheikh University, Kafr El-sheikh, Egypt; e Alfaisal University, Riyadh, Saudi Arabia; f Shadan Institute of Medical Sciences, Hyderabad, India; g Addis Ababa University, Ethiopia; h Jawaharlal Institute of Postgraduate Medical Education and Research (JIPMER), Pondicherry, India; i Al-Azhar University, Cairo, Egypt; j Tanta University, Tanta, Egypt; k Jacobi Medical Centre, Albert Einstein College of Medicine, New York, USA; l Director of Intensive Care Unit, Texas Health Rockwall Hospital, TX USA; m Lebanese University, Beirut, Lebanon; n Zahraa Hospital, Beirut, Lebanon; o SUNY Upstate Medical University, NY, USA

**Keywords:** extracorporeal membrane oxygenation, myocarditis

## Abstract

**Background:**

Fulminant myocarditis (FM) is a potentially life-threatening disease that requires emergency care. The authors’ study aims to explore clinical outcomes and predictors of survival when using veno-arterial extracorporeal membrane oxygenation (VA-ECMO) support for the treatment of FM in adult and pediatric patients to analyze differences between both populations.

**Methods:**

PubMed, Scopus, Web of Science, and Cochrane databases were searched for studies reporting the effect of VA-ECMO on patients diagnosed with fulminant myocarditis. Statistical analysis was performed using R version 4.2.2.

**Results:**

Forty-three studies were included in our analysis with a total of 1268 patients. Survival rates were 65% and 71% among adult and pediatric patients, respectively. Patients who didn’t suffer from cardiac arrest prior to VA-ECMO had better chances of survival in both populations; adults (OR 0.44; *P*<0.01) and pediatric (OR = 0.32; *P*= 0.006). Younger age was associated with higher survival among the adults (MD= −8.81; *P*<0.01). Additionally, pre-ECMO LVEF was higher among survivors in the pediatric group (MD= 8.23; *P*<0.01). Furthermore, no significant association was detected between sex, VA-ECMO duration, systolic blood pressure, lactate levels, and survival rates among both groups.

**Conclusion:**

Using VA-ECMO in patients with fulminant myocarditis can significantly improve survival outcomes, with improved prognosis observed with younger age among adults and absence of prior history of cardiac arrest in both groups.

## Introduction

Highlights

- Using VA-ECMO in patients with fulminant myocarditis can significantly improve survival outcomes.
- Younger adults were more likely to survive, while mean age was not significant among patients in the pediatric group.
- Patients who suffered from cardiac arrest prior to VA-ECMO had a worse prognosis, while lactate and VA-ECMO duration had no effect on survival rates in both populations.
- Higher survival rates were detected among pediatric patients with higher pre-ECMO LVEF.

Fulminant myocarditis (FM) is an inflammatory disease of the myocardium with a sudden and severe onset^1^. In the acute phase, FM can lead to life-threatening arrhythmias and shock, which necessitates emergent care via vasopressors, intra-aortic balloon pumps (IABPs), left ventricular assist devices (LVADs), and mechanical circulatory support^2^. Once the patient survives the acute phase of FM, they have a high chance of survival, given that FM is a self-resolving disease^3^.

Recently, physicians have been leaning toward mechanical circulatory support in the form of veno-arterial extracorporeal membrane oxygenation (VA-ECMO) for the treatment of acute fulminant myocarditis. Studies have shown that it improves survival and is associated with better prognosis among patients suffering from FM^4^. Extracorporeal membrane oxygenation (ECMO) is a procedure that involves removing venous blood, typically from the femoral or internal jugular vein, which is then oxygenated and warmed before being returned to the body via an artificial pump. In VA-ECMO, the blood is pumped back into the arterial circuit. As a result, the blood bypasses the lungs and heart, with the ECMO machine acting as a temporary pump. This allows the lungs and heart to recuperate while maintaining oxygenation to the organs during states of cardiopulmonary compromise. This contrasts with veno-venous ECMO (V-V ECMO), which returns blood back to the venous system and is used in states of respiratory failure^5,6^.

It is important to highlight that FM is a disease that affects both the pediatric and adult populations. While previous articles have analyzed the prognostic value of VA-ECMO in each of these populations individually^7,8^, this study aims to compare both populations, analyze any disparities among both groups and assess possible predictors of survival.

## Methods

This systematic review and meta-analysis were performed and reported following the Cochrane Collaboration Handbook for Systematic Reviews of Interventions and the Preferred Reporting Items for Systematic Reviews and Meta-Analysis (PRISMA) Statement and the AMSTAR guidelines^9^; Supplementary Methods S1-2, Supplemental Digital Content 1, http://links.lww.com/MS9/A613.

### Search strategy

A systematic literature search of the following databases (PubMed, Scopus, Web of Science, and Cochrane) conducted on the 21 August 2022, using key terms such as “ECMO” or “extracorporeal membrane oxygenation”, “ECLS” or “extracorporeal life support”, “cardiac shock” or “cardiogenic shock”, “cardiopulmonary resuscitation” or “cardiac arrest”, fulminant and myocarditis, was performed to identify studies of interest. The complete search strategy is provided in Supplementary Methods S3, Supplemental Digital Content 1, http://links.lww.com/MS9/A613.

### Study eligibility

Inclusion criteria: Controlled observational, cross-sectional, case-control, case-series and cohort studies providing in-hospital outcomes reporting the survival of adult or pediatric patients diagnosed with fulminant myocarditis supported by VA-ECMO were included.

There were no restrictions placed on sex, race, ethnicity, country of origin, or publication date.

Exclusion criteria: Conference/poster abstracts, editorials, case reports/series of less than or equal to 5 cases, review articles, meta-analyses and studies that did not divide patients to adult and/or pediatric groups were excluded.

Two independent reviewers (Y.E.D. and A.A.) screened the studies according to our criteria. A third independent reviewer was consulted to resolve the conflict if consensus was not achieved.

### Data extraction

Two independent reviewers (M.D. and R.W.M.) independently extracted the following data: enrollment period, inclusion and exclusion criteria, follow-up period, baseline patient characteristics, endpoint data. Disagreements were resolved in a panel discussion with the senior author (Y.H.). Baseline characteristics were reported as the mean (±standard deviation) for continuous variables and as percentages for binary variables.

For the baseline data, we extracted the following: last name of the first author, year of publication, study design, sample size, country, age, sex, comorbidities, diagnosis of FM, medications, and conclusion.

For outcomes, the following was extracted: Survival to discharge, sex, the incidence of cardiac arrest before VA-ECMO initiation, age, time from hospital/ICU admission to the initiation of VA-ECMO, VA-ECMO duration, lactate, systolic blood pressure, and pre-ECMO left ventricular ejection fraction.

### Statistical analysis

We summarized binary endpoints using the Mantel–Haenszel random-effects model, with odds ratio (OR) and 95% CI as a measure of effect size for binary endpoints and mean difference for continuous variables. The DerSimonian and Laird (DL) method was used to calculate heterogeneity variance tau². Heterogeneity was assessed with Cochrane’s Jackson method’s *I*² statistics, with *p* less than or equal to 0.10 indicating statistical significance. The consistency of the studies was determined based on *I*² values of 0%, less than or equal to 25%, less than or equal to 50%, and greater than 50%, indicating no observed, low, moderate, and substantial heterogeneity, respectively. In tests that were two-tailed, a *p* value of less than 0.05 was considered statistically significant. Single-armed meta-analysis was done using the inverse variance method with the arcsine or logit transformation, and proportions were reported as a measure for prevalence. We utilized R version 4.2.2 (R Foundation for Statistical Computing) and the extension packages “meta” and “dmetar” for all calculations and graphics^10–12^.

We addressed heterogeneity in heterogeneous outcomes with leave-one-out sensitivity analysis. Heterogeneity was assessed using R² statistics, and a two-tailed *p* value of less than 0.05 indicates statistical significance.

### Quality assessment

For observational studies, the Newcastle–Ottawa scale (NOS) was used to assess the quality of each study included^13^. This scale comprises eight items within three domains: selection, comparability, and outcome/exposure. The NOS assigns a maximum of four points for selection, two for comparability, and three for outcome/exposure. A composite score was calculated to reflect the overall quality of each study, with scores greater than 7 indicating high quality.

Case series studies were assessed using the Joanna Briggs Institute (JBI) Critical Appraisal Tool^14^. When evaluating case series using the JBI critical appraisal tool, studies were judged as having a ‘low risk of bias’ if at least 8 items on the checklist scored ‘yes’; ‘some concerns of bias’ if 5–7 items scored ‘yes’; and a ‘high risk of bias’ if up to five items scored ‘yes’.

Publication bias in outcomes with 10 studies or more was assessed by funnel-plot analysis of point estimates according to study weights and asymmetry was tested by Egger’s regression test.

## Results

### Literature search

**Figure 1:**
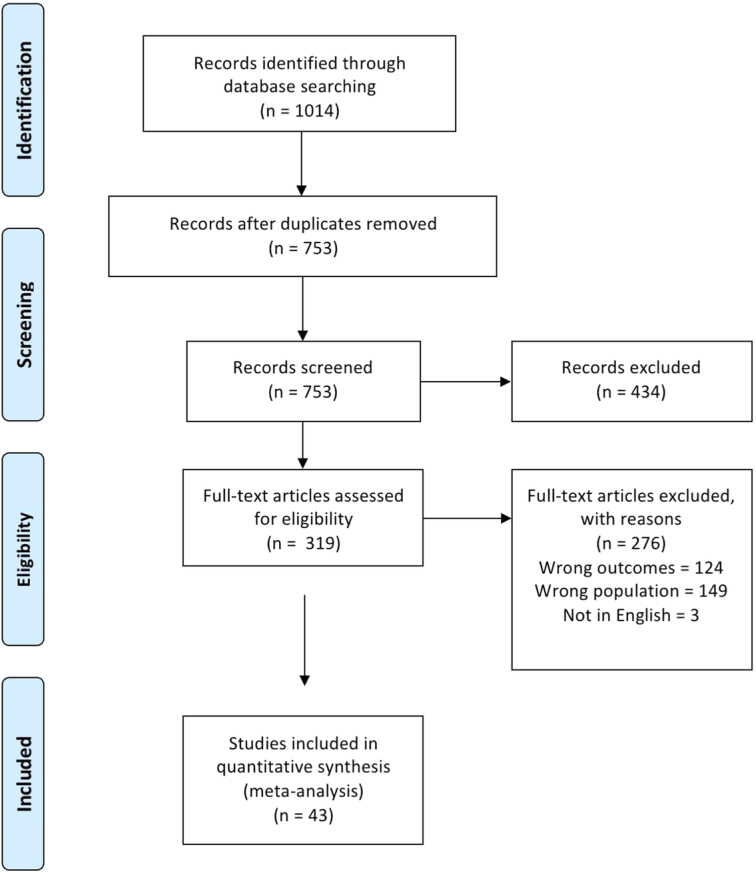
PRISMA. PRISMA, Preferred Reporting Items for Systematic Reviews and Meta-Analysis

The initial search yielded 1014 studies. After removing duplicates, 753 studies were retrieved that were eligible for the title and abstract screening. Of the 753, 434 were irrelevant, with 319 studies remaining eligible for full-text screening. Finally, 43^15–57^ studies were included in the final meta-analysis after the full-text screening, as shown in Fig. 1.

The total number of patients included in the study is 1268; 722 patients in the adult and 546 patients in the pediatric group; other baseline characteristics are reported in Table 1.

**Table 1:** Baseline Characteristics of included studies.

Author (year)	Population	Study design	Country	Age (years), mean (SD)	Sex (males), *n* (%)	SBP, mean (SD)	ECMO duration (days), mean (SD)
Duncan *et al*. (2001)^29^	Pediatric	Case series	USA & Canada	5.7 (3.98)	8 (53.3)	—	—
Lee *et al*. (2020)^22^	Pediatric	Retrospective cohort	Taiwan	8.82 (6.09)	13 (39.4)	88.8 (28.03)	5.66 (3.69)
Lee *et al*. (2014)^15^	Pediatric	Retrospective cohort	South Korea	5.71 (3.46)	3 (17.6)	—	7.15 (2.13)
Mani *et al*. (2010)^16^	Pediatric	Case series	Singapore	6.75 (2.63)	3 (37.5)	—	8.42 (5.14)
Nahum *et al*. (2010)^30^	Pediatric	Case series	Israel	2.125 (2.47)	5 (41.7)	—	8.75 (3.29)
Okada *et al*. (2016)^17^	Pediatric	Case series	Japan	7.03 (4.48)	5 (62.5)	—	5.25 (2.63)
Schubert *et al*. (2019)^23^	Pediatric	Prospective cohort	Germany	—	—	—	—
Şik *et al*. (2019)^31^	Pediatric	Case series	Turkey	5.25 (3.79)	3 (50)	53.83 (29.58)	6.8 (3.36)
Sun *et al*. (2022)^28^	Pediatric	Retrospective cohort	China	8 (3.42)	17 (41.5)	—	—
Teele *et al*. (2011)^24^	Pediatric	Retrospective cohort	USA	10.91 (5.09)	11 (55)	—	—
Tuan *et al*. (2021)^20^	Pediatric	Retrospective cohort study	Vietnam	6.67 (5.33)	25 (44)	88.6 (20.05)	7.8 (5.01)
Wilmot *et al*. (2011)^18^	Pediatric	Retrospective cohort	USA	7.01 (3.99)	5 (31.3)	—	6.05 (3.24)
Wu *et al*. (2006)^19^	Pediatric	Retrospective cohort	Taiwan	9.1(3.3)	3 (33.3)	—	4.8 (1.4)
Wu *et al*. (2017)^21^	Pediatric	Retrospective cohort	Taiwan	8.8 (6.32)	32 (53.3)	—	—
Ammirati *et al*. (2017)^50^	Adult	Retrospective cohort	Italy	35.86 (12.93)	7 (46.6)	—	—
Ammirati *et al*. (2017)^50^	Pediatric	Retrospective cohort	Italy	7.78 (6.32)	0 (0)	—	—
Ang *et al*. (2008)^46^	Adult	Retrospective cohort	Singapore	45.67 (42.2)	31 (75.6)	—	—
Asaumi *et al*. (2005)^47^	Adult	Retrospective cohort	Japan	38 (15)	7 (50)	74 (15)	4.76 (4.42)
Chen *et al*. (2005)^27^	Adult	Case-control	Taiwan	41.07 (15.51)	—	61.46 (6.46)	5.48 (2.62)
Chen *et al*. (2005)^27^	Pediatric	Case-control	Taiwan	5.93 (4.34)	—	58.63 (14.83)	5.226 (3.03)
Chong *et al*. (2018)^34^	Adult	retrospective cohort	Taiwan	40.7 (14.7)	18 (51.4)	76.02 (38.48)	7.82 (5.39)
Chou *et al*. (2020)^41^	Adult	Retrospective cohort	Taiwan	41.8 (14.5)	35 (39.7)	—	9.4 (8.6)
Hsu *et al*. (2011)^25^	Both	Retrospective cohort	Taiwan	29.7 (18.7)	29 (38.6)	75.5 (26.7)	7.14 (4.98)
Hu *et al*. (2018)^36^	Adult	Case series	China	31.2 (14.6)	4 (57.1)	—	1.949 (1.901)
Ishida *et al*. (2011)^43^	Adult	Case series	Japan	41.87 (17.39)	12 (60)	82.67(24.62)	—
Jung *et al*. (2016)^32^	Pediatric	Retrospective cohort	South Korea	3.52 (3.65)	5 (38.5)	—	6.55 (3.28)
Lee *et al*. (2021)^26^	Both	Retrospective cohort	Korea	29.96 (25.96)	44 (44)	—	—
Lee *et al*. (2021)^26^	Adult	Retrospective cohort	Korea	42.02 (14.19)	—	—	—
Lee *et al*. (2021)^26^	Pediatric	Retrospective cohort	Korea	5 (6.91)	—	—	—
Li (2020)^53^	Adult	Retrospective cohort	China	—	—	—	—
Liao *et al*. (2017)^35^	Adult	Retrospective cohort	China	33.0 (19.0)	16 (48.4)	—	3 (1.83)
Lin *et al*. (2013)^33^	Pediatric	Prospective cohort study	Taiwan	—	—	—	—
Lorusso *et al*. (2016)^38^	Adult	Retrospective cohort	Italy	37.6 (11.8)	20 (35.1)	61.8 (30.4)	9.9 (19)
Martinez-Solano (2021)	Adult	Cohort study	Spain	56.3 (12.1)	93 (76.9)	—	—
Manabu Matsumoto *et al*. (2018)^48^	Adult	Retrospective cohort	Japan	42.22 (30.15)	21 (56.7)	82.77 (15.6)	6.7 (4.7)
Mirabel *et al*. (2011)^45^	Adult	Retrospective cohort	France	—	—	—	—
Montero *et al*. (2018)^49^	Adult	Cohort study	France	45.99 (16.94)	5 (45.4)		12.78 (10.04)
Nakamura *et al*. (2015)^42^	Adult	Retrospective cohort	Japan	46.2 (18.7)	10 (45.4)	83.2 (23.6)	7.47 (1.06)
Saito *et al*. (2017)^54^	Adult	Retrospective cohort	Japan	—	—	—	4.67 (9.48)
Sawamura *et al*. (2018)^40^	Adult	Retrospective cohort	Japan	53 (16)	52 (59.8)	—	6.67 (4.24)
Seguchi *et al*. (2017)^52^	Adult	Retrospective cohort	Japan	—	—	—	—
Ting *et al*. (2016)^44^	Both	Retrospective cohort	Taiwan	—	—	—	—
Ting *et al*. (2016)^44^	Adult	Retrospective cohort	Taiwan	38.57 (18.2)	33 (37.5)	87.42 (24.22)	7.02 (3.97)
Ting *et al*. (2016)^44^	Pediatric	Retrospective cohort	Taiwan	—	—	—	—
Zuo *et al*. (2019)^56^	Adult	Prospective	China	34 (18)	9 (45)	91 (11)	—
Weymann *et al*. (2014)^57^	Both	Prospective	Germany	23 (8.71)	5 (83.3)	—	9.83 (5.11)
Weymann *et al*. (2014^57^)	Adult	Prospective	Germany	27.75 (6.72)	3 (75)	—	12 (5)
Weymann *et al*. (2014)^57^	Pediatric	Prospective	Germany	13.5 (1.5)	2 (100)	—	5.5 (0.5)
Bhardwaj *et al*. (2022)^51^	Adult	Case series	USA	40(17.49)	5 (55.6)	—	6.78 (2.39)
Ho *et al*. (2022)^37^	Adult	Retrospective cohort	Taiwan	48.2 (13.3)	13 (46.4)	—	8.32 (6.75)
Wang *et al*. (2022)^39^	Adult	Retrospective cohort	Taiwan	42.7 (17.7)	13 (59.1)	—	—

SBP, systolic blood pressure.

### Outcomes

Pooled analysis of included studies is reported in Table 2.

**Table 2:** Pooled analysis of included studies.

(A) Prevalence of survival to discharge in included studies.				
Group	Proportion	95% CI	*I* ^2^	
Adults	65.3	[60.9; 69.4]	12%	
Pediatrics	71.3	[66.4; 75.7]	0%	
(B) Odds of survival (binary data).				
	Odds ratio	95% CI	*I²*	*p*
Adults				
Sex (males)	1.0	[0.7; 1.5]	0%	0.871
Cardiac arrest	0.4	[0.2; 0.9]	37%	**0.021**
Pediatrics				
Sex (males)	1.4	[0.5; 3.9]	3%	0.573
Cardiac arrest	0.3	[0.2; 0.7]	0%	**0.006**
(C) Predictors of survival—survivors vs non-survivors (continuous data).				
	Mean difference	95% CI	*I²*	*p*
Adults				
Mean age	−8.8	[−14.2; −3.5]	59%	**<0.01**
Time from hospital admission to the initiation of ECMO	−17	[−40.1; 6.1]	88%	0.15
ECMO duration	−1.4	[−3.1; 0.3]	80%	0.11
Lactate	−6.1	[−12.96; 0.8]	77%	0.08
Systolic Blood pressure	3.6	[−0.4; 7.7]	0%	0.08
Pre-ECMO LVEF	−3.6	[−8.2; 0.98]	47%	0.12
Pediatrics				
Mean age	1.1	[−0.3; 2.4]	0%	0.12
Time from hospital admission to the initiation of ECMO	−11.8	[−41.1; 17.4]	58%	0.43
ECMO duration	−0.5	[−2.6; 1.7]	50%	0.67
Lactate	−2.2	[−6.5 2.2]	63%	0.33
Systolic blood pressure	6.4	[−6.9; 19.8]	0%	0.34
Pre-ECMO LVEF	8.2	[2.4; 14.1]	0%	**<0.01**

ECMO, extracorporeal membrane oxygenation; LVEF, left ventricular ejection fraction.

### Prevalence of survival to discharge

**Figure 2:**
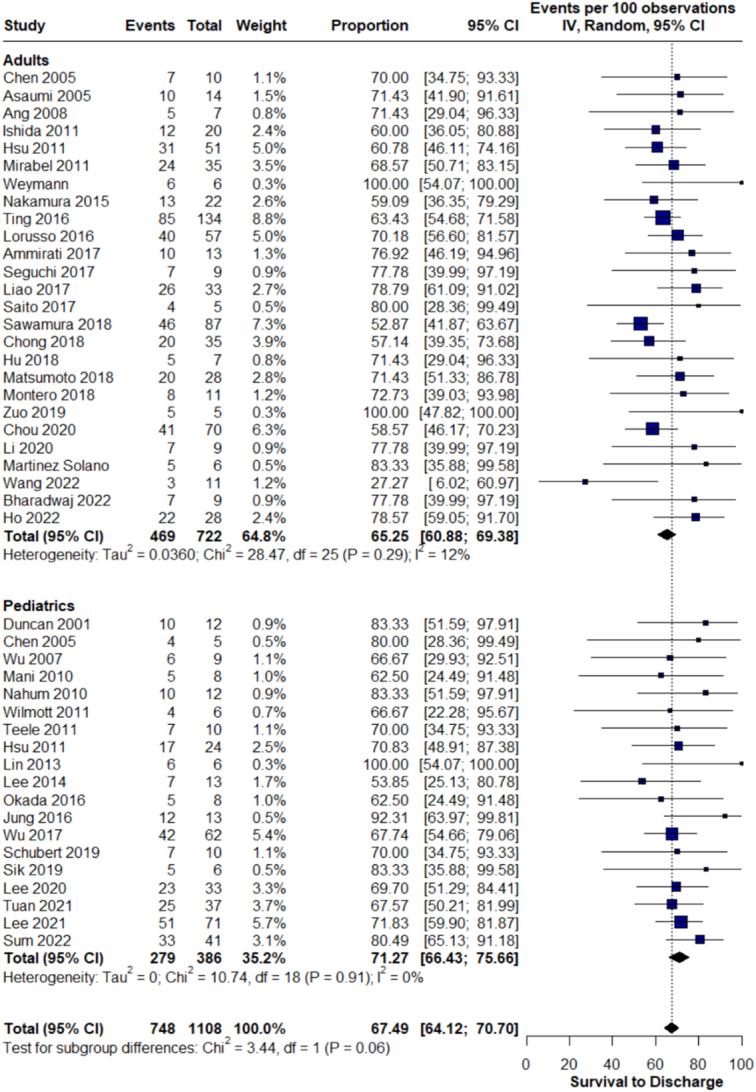
Survival to discharge.

In the adult population, the survival to discharge was 65.2% (95% CI 61.2–69.4%; *I*²=12%; Fig. 2A), while the pediatric population had a higher survival rate of 71.3% (95% CI: 66.4–75.7%; *I*²=0%, the overall survival to discharge was 67.5%; Fig. 2B).

### Survival odds

#### Sex

**Figure 3:**
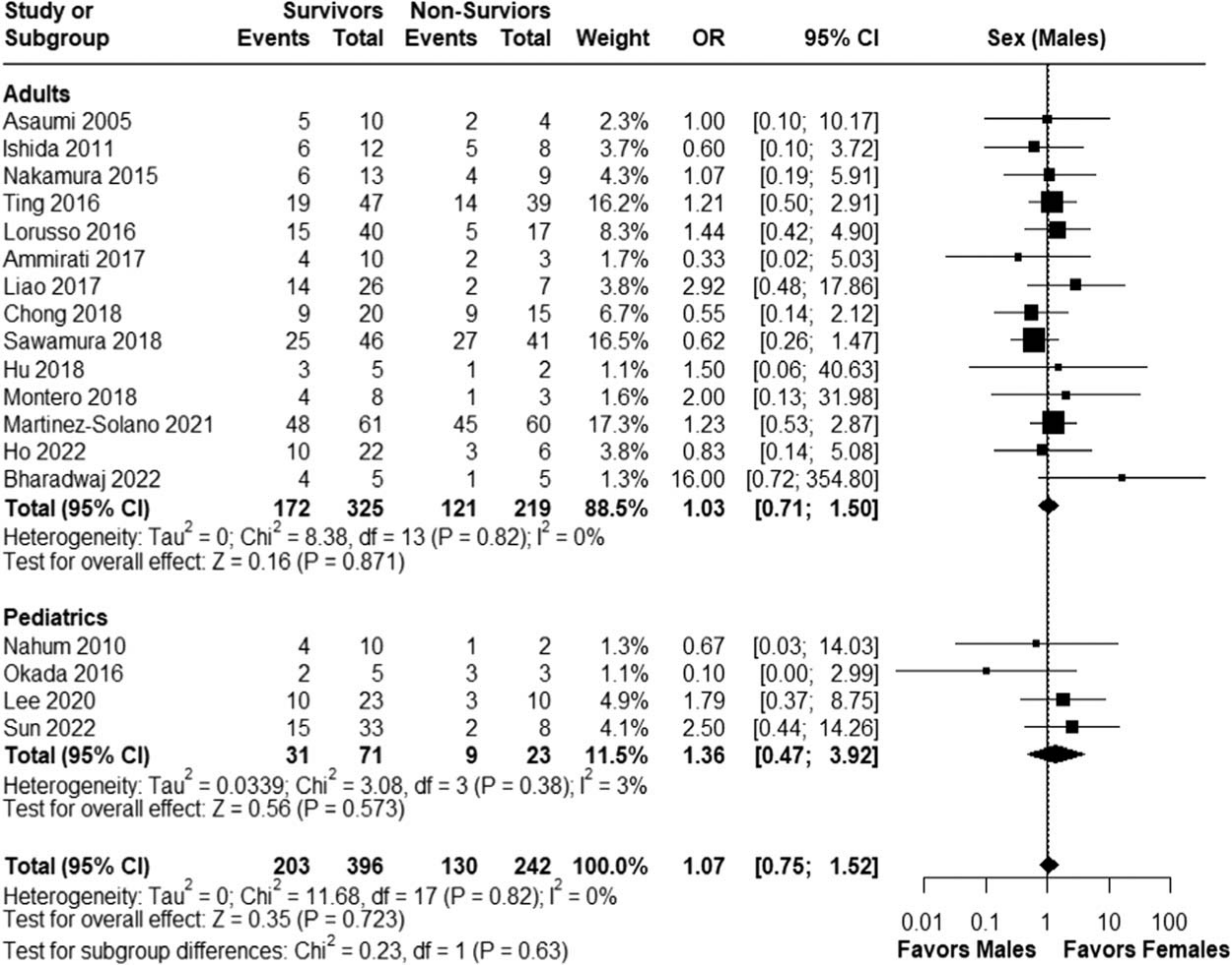
Sex (males); survivors versus non-survivors. OR, odds ratio.

There was no statistically significant association between male gender and survival to discharge in the adults (OR 1.03; 95% CI 0.71–1.50; *P* = 0.87; *I*²=0%) and pediatric populations (OR: 1.36; 95% CI 0.47–3.92; *P*= 0.57; *I²*=3%), overall effect size was (OR 1.07; 95% CI 0.75; 1.52; *P*=0.72; *I²*=0%); Fig. 3.

#### Cardiac arrest

**Figure 4:**
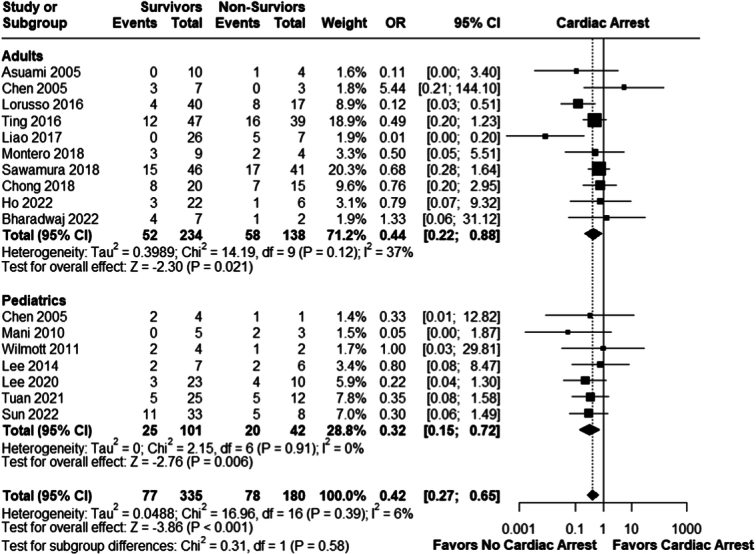
Cardiac arrest; survivors versus non-survivors. OR, odds ratio.

Patients who didn’t suffer from cardiac arrest prior to ECMO initiation had better chances of survival in both populations; adults (OR 0.44; 95% CI 0.22–0.88; *P* = 0.002; *I²*=37%) and pediatric (OR= 0.32, 95% CI: 0.15–0.72; *P* = 0.006; *I²*=0%), overall effect size was ( OR 0.42; 95% CI 0.27–0.65; *P*<0.001; *I²*=6%); Fig. 4.

### Predictors of survival

#### Age

**Figure 5 (A):**
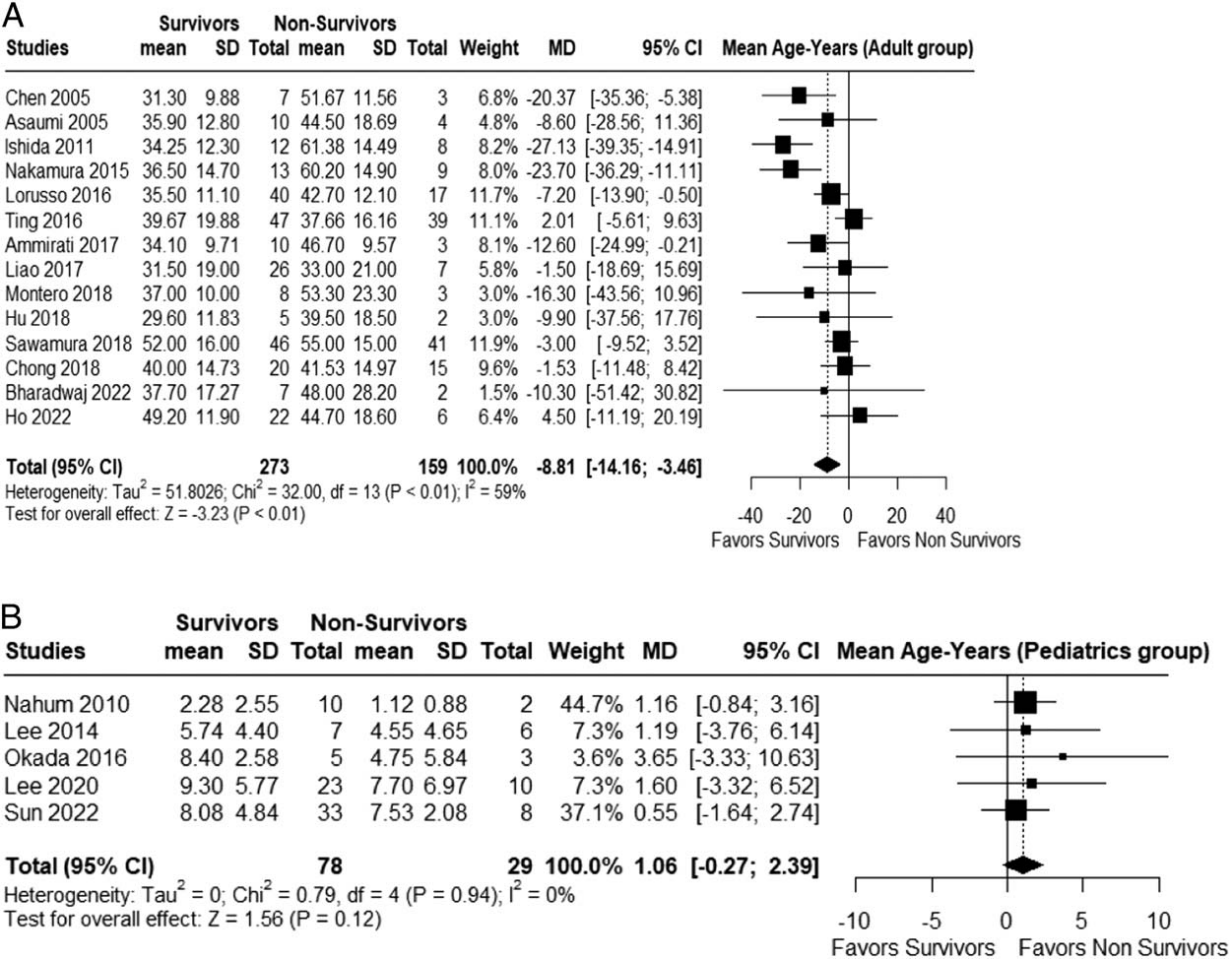
Age; adult population. (B) Age; pediatric population.

In the adult population, younger patients had significantly higher survival rates (MD –8.81; 95% CI −14.16 to -3.46; *P*<0.01; *I²*=59%; Fig. 5A). However, our analysis showed no significant difference in mean age between survivors and non-survivors in the pediatric population (MD 1.06; 95% CI −0.27 to 2.39; *P*= 0.12; *I²*=0%; Fig. 5B).

#### Time from hospital admission to the initiation of VA-ECMO

**Figure 6:**
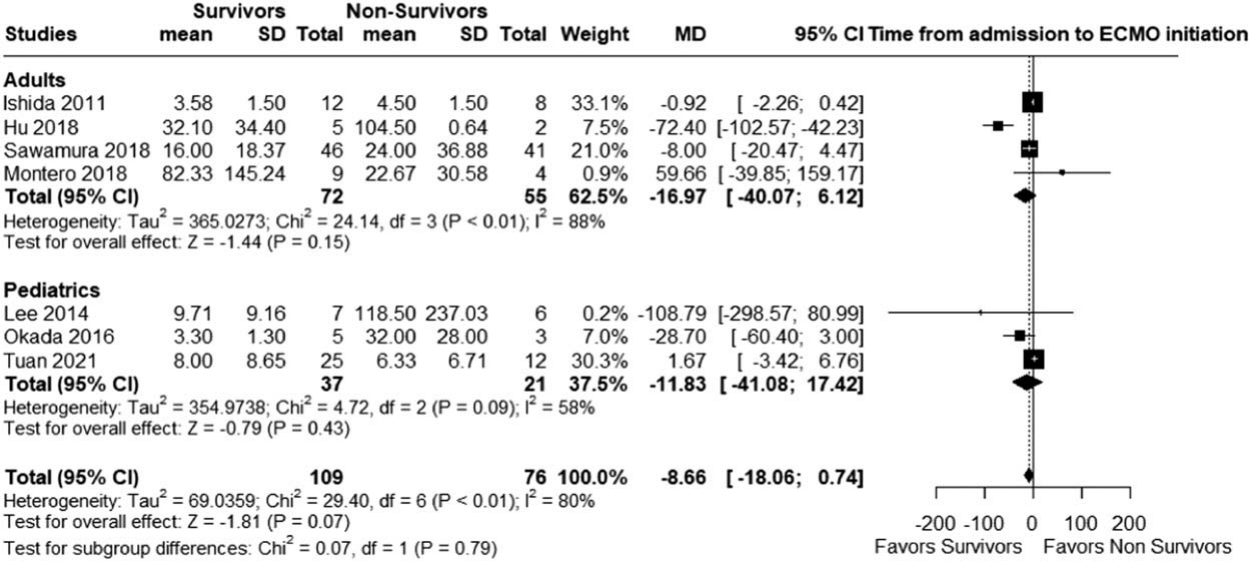
The Time from hospital admission to the initiation of ECMO. ECMO, extracorporeal membrane oxygenation.

There was no statistically significantly difference between survivors and non-survivors when it came to delay between hospital admission to initiation of ECMO; adults (MD −16.97; 95% CI −40.07 to 6.12; *P*=0.15; *I²*=88%) and pediatric (MD 11.83; 95% CI:−41.08 to 17.42; *P*=0.43; *I²*=58%), the overall mean difference was (MD 8.66; 95% CI −18.06 to 0.74; *P*=0.07; *I²*=80%); Fig. 6.

#### ECMO duration

**Figure 7:**
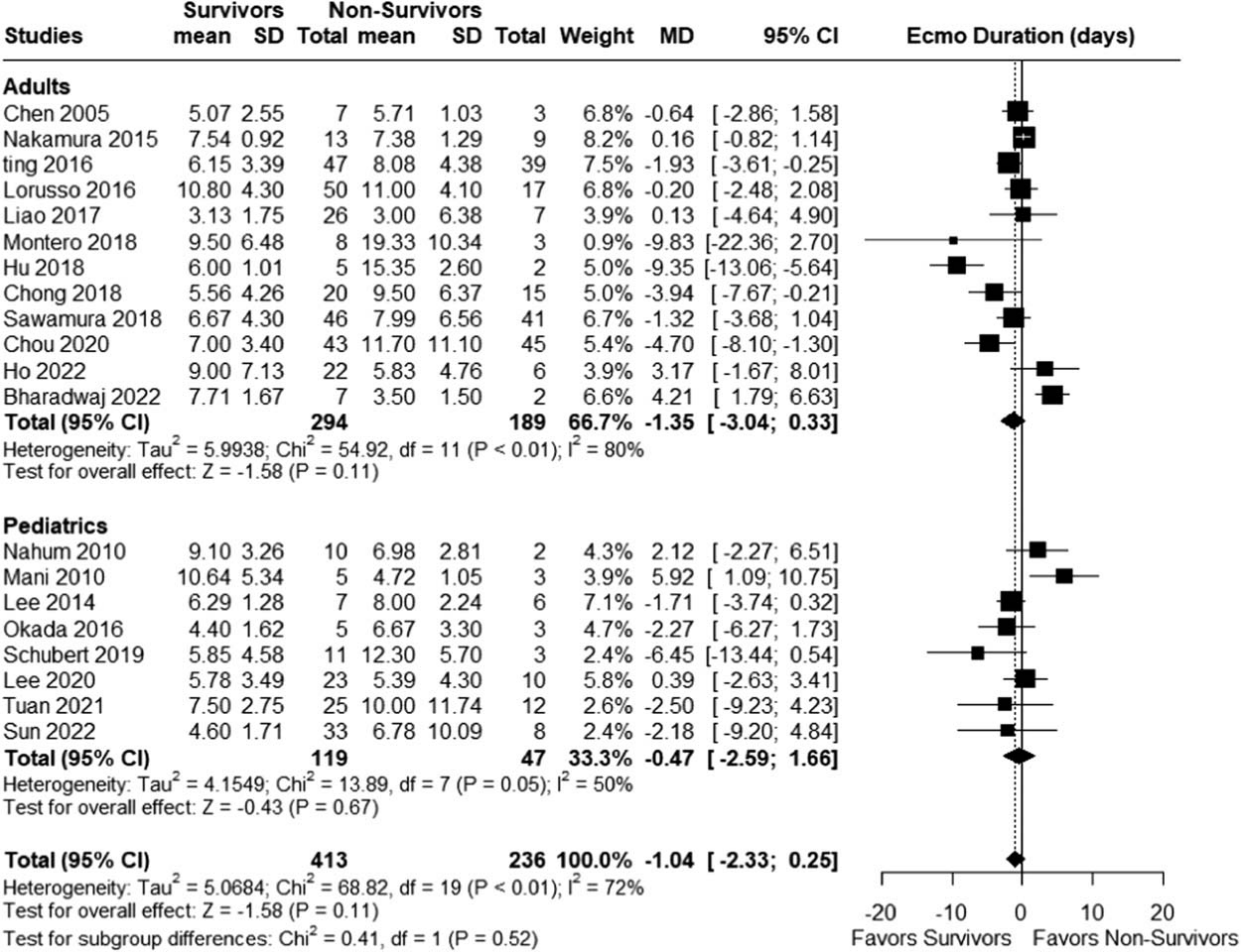
Mean ECMO duration (days). ECMO, extracorporeal membrane oxygenation.

There was no statistically significant difference in mean duration of ECMO in days between survivors and non-survivors in both populations; adults (MD −1.35, 95% CI −3.04 to 0.33; *P*=0.11; *I²*=80%) and pediatric (MD: −0.47, 95% CI: −2.59 to 1.66; *P*=0.67), overall mean difference was (MD −1.04; 95% CI −2.33 to 0.25; *P*=0.11; *I²*=72%); Fig. 7.

#### Mean serum lactate

**Figure 8:**
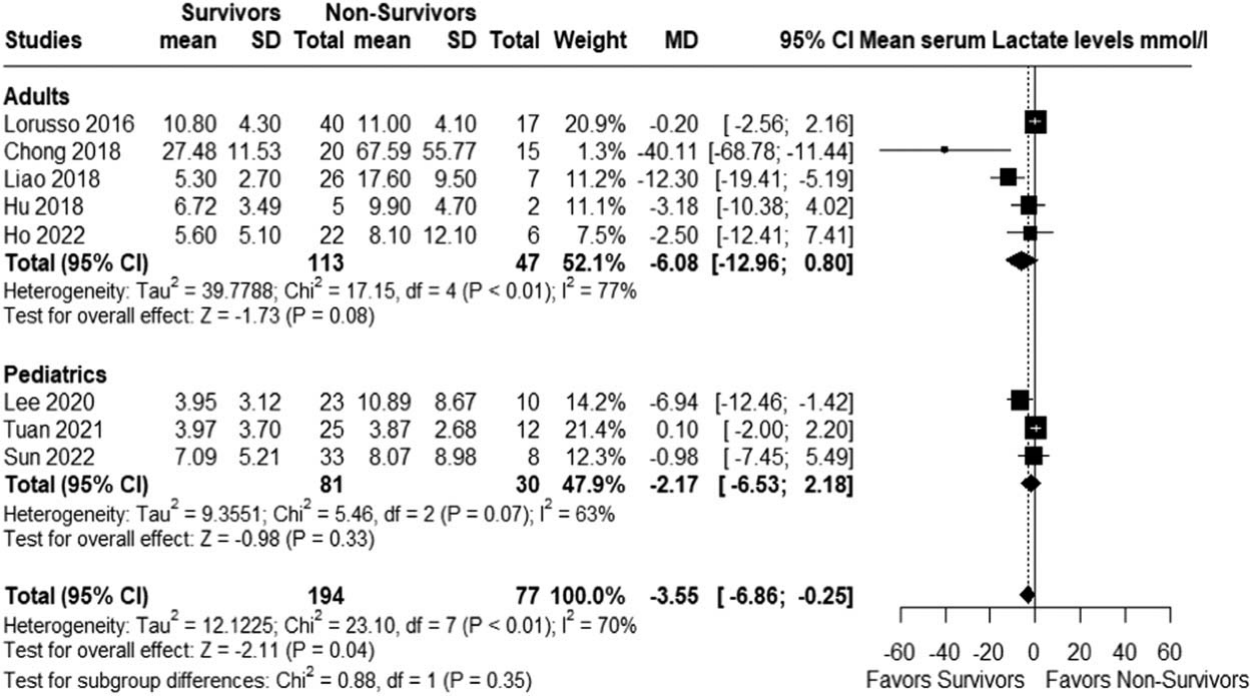
Mean serum lactate levels mmol/l.

Serum levels of lactate (mmol/l) were not statistically different between survivors and non-survivors on ECMO; adults (MD −6.08 (95% CI: −12.96 to 0.80; *P*=0.08; *I²*=77%) and pediatric (MD −2.17 (95% CI: −6.53 to 2.18; *P*=0.33; *I²*=63%). However, the overall mean difference was statistically significantly lower in the survivors’ group (MD −3.55; 95% CI −6.86 to −0.25; *P*= 0.04; *I²*=70%); Fig. 8.

#### Systolic blood pressure (SBP)

**Figure 9:**
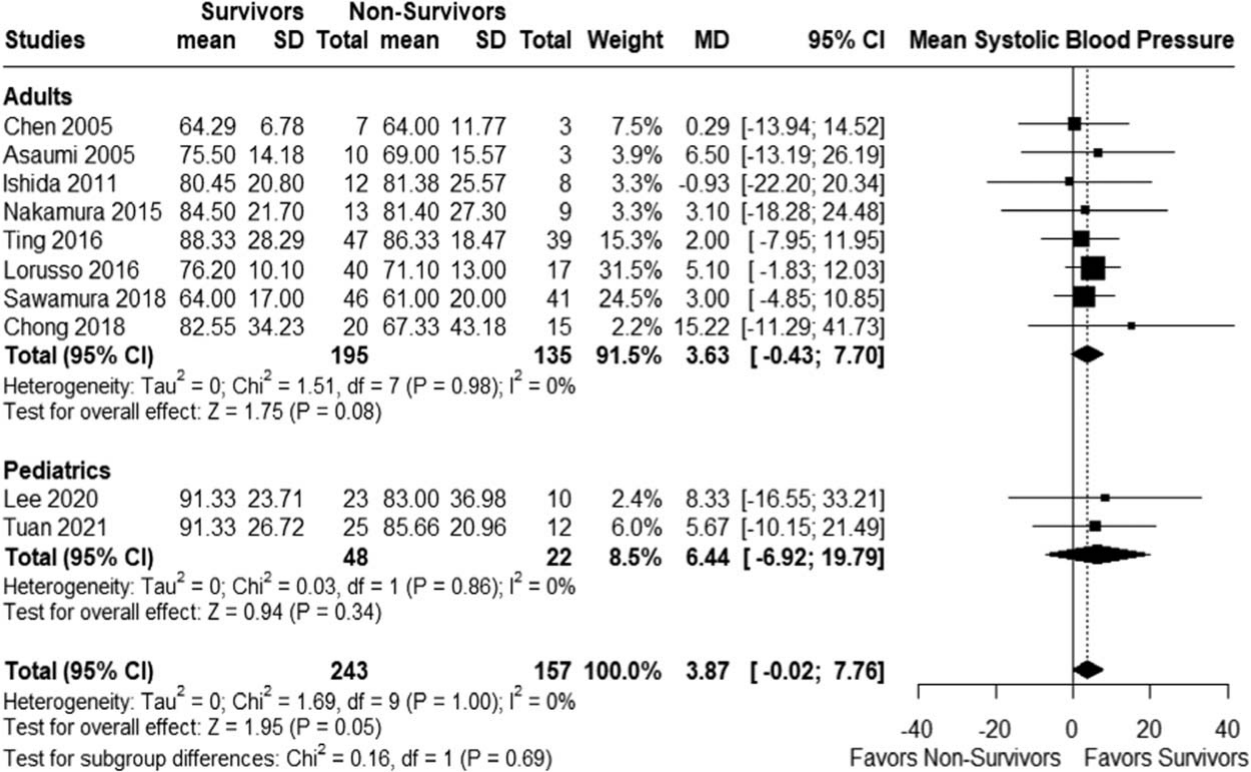
Mean systolic blood pressure mm/hg..

The mean SBP was not statistically significant between survivors and non-survivors in both groups; adults (MD 3.63; 95% CI −0.43 to 7.70; *P*=0.08; *I²*= 0%); pediatric group (MD 6.44; 95% CI −6.92 to 19.79; *P*=0.34; *I²*=0%), the overall mean difference also showed no statistically significant difference (MD 3.87; 95% CI −0.02 to 7.76; *P*=0.05; *I²*=0%); Fig. 9.

#### Pre-ECMO left ventricular ejection fraction (LVEF)

**Figure 10:**
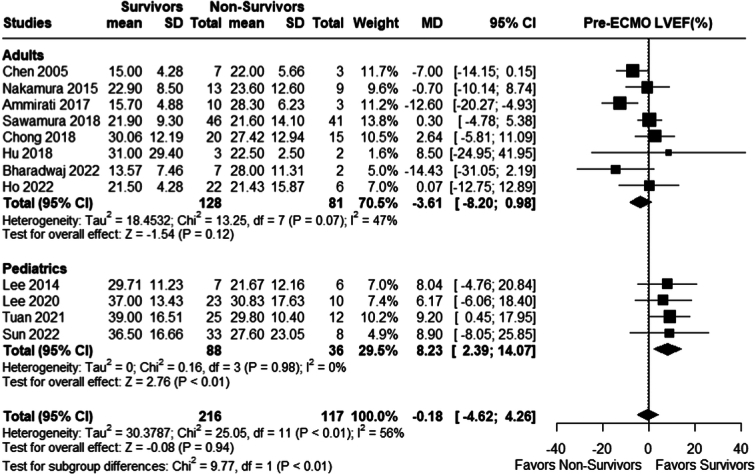
Pre-ECMO left ventricular ejection fraction (LVEF). ECMO, Extracorporeal membrane oxygenation.

There was no significant difference in pre-ECMO LVEF among survivors and non-survivors in the adults (MD −3.61; 95% CI −8.20 to −0.98; *P*=0.12; *I²*=47%). However, the pediatric group showed a higher pre-ECMO LVEF in the survivors (MD 8.23, 95% CI: 2.39 to 14.07; *P*< 0.01; *I²*=0%), while the overall mean difference remained insignificant (MD −0.18; 95% CI −4.62 to 4.26; *P*=0.94; *I²*=56%); Fig. 10.

#### Sensitivity analysis

We explored the consistency of treatment effects using the leave-one-out strategy, which helped in the detection of studies contributing to the heterogeneity in each reported outcome (Supplementary Figures S1, Supplemental Digital Content 1, http://links.lww.com/MS9/A613). While heterogeneity was not completely resolved using this method in outcomes such as mean age, time from hospital admission to ECMO duration, mean serum lactate and pre-ECMO LVEF, the residual heterogeneity was attributed to the differences in baseline characteristics between groups due to the non-randomized nature of selected studies. Furthermore, the leave-one-out analysis did not reveal any significant changes in outcomes when any individual study was removed from the analysis across all heterogeneous outcomes examined.

### Quality assessment

Individual appraisal of Cohort and case-controlled studies is reported in Supplementary Tables S1 and S2, Supplemental Digital Content 1, http://links.lww.com/MS9/A613. Twenty-seven cohort studies were rated as low quality due to inadequate definition of case and controls, while 9 studies were given a score of 7 or higher deeming them high-quality studies. All 6 case series studies were defined as low risk of bias as 8 or more items on the checklist scored ‘yes’. Funnel plot analysis and Egger regression test detected no evidence of publication bias for the included studies (Supplementary Figures S2, Supplemental Digital Content 1, http://links.lww.com/MS9/A613 and Supplementary Tables S3, Supplemental Digital Content 1, http://links.lww.com/MS9/A613).

## Discussion

Regarding the current literature, there are not many clinical trials evaluating the prognosis of patients with FM under VA-ECMO support, especially when it comes to a comparison between adult and pediatric populations. Our results showed that the survival rate for adult patients treated with ECMO for FM was 65.3%, while pediatric patients had a survival rate of 71.3%. Younger adults had higher survival rates, while age did not impact pediatric patients. Both adults and pediatric patients without a previous history of cardiac arrest had superior survival odds. Pre-ECMO LVEF was higher in survivors among the pediatric group; however, no significant difference was observed between survivors and non-survivors in the adult group. No significant difference was reported for other factors such as sex, time from admission to initiation of ECMO, ECMO duration, lactate levels or systolic blood pressure.

The presence of a cardiac arrest event before the initiation of VA-ECMO did have substantial ramifications in terms of survival rates, with the absence of cardiac arrest being associated with a better prognosis. These findings are similar to those of Schlapbach *et al.* ^58^, who demonstrated in their multi-centric cohort study conducted on children that the absence of cardiac arrest prior to VA-ECMO predicted significantly higher survival among children undergoing VA-ECMO for sepsis. Similar findings were detected among adults, as reported by Zhang *et al* 2021 in their review^59^.

Age was found to be a major prognostic factor in the adult population, as previously noted. Younger age in the adult population was linked to higher survival rates. Vishram-Nielsen and colleagues supported our findings, concluding that younger patients had lower mortality rates. Their meta-analysis included only adults, with a mean age of 40^7^. Chong *et al* ^34^ conversely concluded that older age isn’t a risk factor for higher mortality among the adult population, as they detected an insignificant association between age and survival rates. It is important to note that this study included a small sample size of 35 patients and that among the adult FM patients on VA-ECMO, older patients had a worse prognosis, possibly due to the higher prevalence of comorbidities. Lee *et al.* ^60^ proved this by identifying a significant association between older age and poorer survival rates that disappeared after the adjustment for comorbidities. We concluded from the extracted data of our results that age did not influence survival in pediatric patients undergoing ECMO for FM. This is corroborated by Xiong H.‘s meta-analysis of pediatric patients, where no correlation between age and survival to discharge was noted.

A multitude of factors between the survivor and non-survivor subgroups in both age populations were analyzed. The duration of VA-ECMO did not have a consequential association in either subgroup among the two populations. Similarly, Lorusso *et al.* ^38^’s study showed that the duration of ECMO was similar among adult survivors and non-survivors, with a mean duration of 9.2 and 9 days, respectively.

We also compared the patients’ pre-ECMO systolic blood pressure and left ventricular ejection fraction to determine the effect of their hemodynamic and cardiac function prior to ECMO on survival outcomes. Our results showed that while there was no effect of pre-ECMO SBP on survival in either group, a higher LVEF before initiating VA-ECMO was significantly associated with better survival rates in children. Our findings are consistent with Xie *et al.* ^61^, a study that concluded that LVEF was an independent prognostic factor amongst pediatric FM patients.

Unlike the level of LVEF, gender did not have a discernible difference between survivors and non-survivors in either age population group, reinforcing the findings of Wang *et al.* ^57^, which showed that sex doesn’t play a role in survival among those suffering from cardiogenic shock supported by VA-ECMO. These results oppose a study conducted by Gill *et al.* ^62^, which showed that female patients undergoing VA-ECMO for cardiac arrest had higher survival rates as compared to their male counterparts.

When considering prognostic factors, the significance of lactate levels has been established in patients with shock. However, limited information is available regarding its role as a prognostic factor in patients with fulminant myocarditis undergoing VA-ECMO. Lactate is produced when cells undergo anaerobic metabolism due to inadequate oxygen delivery. Persistently high lactate levels, despite VA-ECMO support, suggest ongoing tissue hypoxia or inadequate cardiac output. In our study, there was no significant statistical difference in lactate levels amongst survivors and non-survivors in the adult and pediatric populations separately, but the pooled analysis showed significantly lower lactate levels in survivors as compared to non-survivors. Previous studies have reported conflicting data when it comes to the effect of pre-ECMO lactate levels upon survival. A study by Merkle-Storms *et al*.^63^ shows that pre-ECMO lactate levels were similar for survivors and non-survivors in pediatric patients. Conversely, a study by Laimoud *et al*.^64^ showed that pre-ECMO lactate levels were significantly lower in survivors as compared to non-survivors among adult patients undergoing VA-ECMO. Individual studies in our synthesis have also documented lactate levels throughout patient treatment, such as those by Lee and colleagues and Matsumoto and colleagues, and a statistical significance between lactate levels and survival was not established in those studies either^26,48^.

### Future directions

This meta-analysis provides adequate evidence for using VA-ECMO as a treatment modality for FM, as well as factors predicting the survival of FM patients undergoing VA-ECMO. Further prospective and randomized studies are warranted to corroborate these findings as well as further analysis to various comorbidities to aid in optimal patient selection criteria for VA-ECMO.

### Strengths and limitations

This study is the first of its kind to investigate in detail the prognostic factors and survival odds of FM patients undergoing VA-ECMO in adult as well as pediatric patients. This led to the control of age as a confounding factor, which may have led to an overestimated effect size if done otherwise. Nonetheless, this study still poses certain limitations. This study includes only observational data. Due to the absence of randomization, each study bears inherent selection bias linked to patients’ individual characteristics. 27 out of possible 36 cohorts were rated “low” quality using the NOS adding potential uncertainty to the results. However, this limitation is mainly attributed to the lack of randomized and prospective data in the literature.

## Conclusion

Using VA-ECMO to treat patients with fulminant myocarditis was associated with good survival rates. Higher survival rates were detected among younger adults and pediatric patients with a higher pre-ECMO LVEF. Patients who suffered from cardiac arrest prior to VA-ECMO had a worse prognosis, while SBP, and VA-ECMO duration did not affect survival rates.

## Ethical approval

Ethics approval was not required for this review and meta-analysis.

## Consent

Informed consent was not required for this review and meta-analysis.

## Source of funding

None.

## Author contribution

Conceptualization: S.S.E. Screening: A.A., N.I., M.Z.M., S.K., H.B. Data extraction: B.A.A.B., A.A., Y.E.D., D.I. Statistical analysis: M.D., S.S.E. Writing: M.D., A.R., M.E., A.M., F.B., Y.E.D., H.A., R.W.M., M.O. Supervision: Y.E., E.D., H.A. All authors reviewed the final manuscript and approved it.

## Conflicts of interest disclosure

All authors declare no conflict of interest

## Research registration unique identifying number (UIN)

http://www.researchregistry.com

reviewregistry1812

https://www.researchregistry.com/browse-the-registry#registryofsystematicreviewsmeta-analyses/

## Guarantor

Yusef Hazimeh.

## Data availability statement

Data are available upon request to the corresponding author.

## Provenance and peer review

My paper wasn’t invited.

## Supplementary Material
