# Supplementary material for: Comparison of predictors of survival among fulminant myocarditis patients undergoing veno-arterial extracorporeal membrane oxygenation in the adult and pediatric populations
Source: Ann Med Surg (Lond). 2024 Oct 16;86(12):7049–61. doi: 10.1097/MS9.0000000000002636 (PMC11623825; doi:10.1097/MS9.0000000000002636)
Supplement: Supplementary file 1 [file ms9-86-7049-s001.docx]

**Comparison of Predictors of Survival among Fulminant Myocarditis Patients Undergoing Veno-arterial Extracorporeal Membrane Oxygenation in the Adult and Pediatric Population.**

**Supplementary Files**

# **Table of contents**

**Supporting Information Methods S1.** PRISMA 2020 Main Checklist 3

**Supporting Information Methods S2.** AMSTAR Checklist 6

**Supporting Information Methods S3**. Details of the Search Strategy 8

**Supporting Information Methods S4**. Addressing Heterogeneity 9

**Supplementary Figures S1**. Leave-out-analysis 9

**Supporting Information Methods S5**. Quality Assessment 13

**Supplementary Table S1:** Quality Assessment for Cohort Studies [1](#_heading=h.tyjcwt)4

**Supplementary Table S2:** Quality Assessment for Case-series [1](#_heading=h.tyjcwt)6

**Supplementary Figures** [**S2.** Publication bias assessment 1](#_heading=h.tyjcwt)7

**Supplementary Tables S3:** Egger’s regression test  [1](#_heading=h.tyjcwt)9

**Supporting Information Methods S1.** PRISMA 2020 Main Checklist

| **Section and Topic** | **Item #** | **Checklist item** | **Location where item is reported** | |
| --- | --- | --- | --- | --- |
| **TITLE** | | |  |  |
| Title | 1 | Identify the report as a systematic review. | Page 1 | |
| **ABSTRACT** | | |  |  |
| Abstract | 2 | See the PRISMA 2020 for Abstracts checklist. | Page 1 | |
| **INTRODUCTION** | | |  |  |
| Rationale | 3 | Describe the rationale for the review in the context of existing knowledge. | Page 2 | |
| Objectives | 4 | Provide an explicit statement of the objectives or questions the review addresses. | Page 2 | |
| **METHODS** | | |  |  |
| Eligibility criteria | 5 | Specify the inclusion and exclusion criteria for the review and how studies were grouped for the syntheses. | Page 3 | |
| Information sources | 6 | Specify all databases, registers, websites, organisations, reference lists and other sources searched or consulted to identify studies. Specify the date when each source was last searched or consulted. | Page 3 | |
| Search strategy | 7 | Present the full search strategies for all databases, registers and websites, including any filters and limits used. | Page 3 | |
| Selection process | 8 | Specify the methods used to decide whether a study met the inclusion criteria of the review, including how many reviewers screened each record and each report retrieved, whether they worked independently, and if applicable, details of automation tools used in the process. | Page 3 | |
| Data collection process | 9 | Specify the methods used to collect data from reports, including how many reviewers collected data from each report, whether they worked independently, any processes for obtaining or confirming data from study investigators, and if applicable, details of automation tools used in the process. | Page 3 | |
| Data items | 10a | List and define all outcomes for which data were sought. Specify whether all results that were compatible with each outcome domain in each study were sought e.g. for all measures, time points, analyses, and if not, the methods used to decide which results to collect. | Pages 3, 4 | |
|  | 10b | List and define all other variables for which data were sought e.g. participant and intervention characteristics, funding sources. Describe any assumptions made about any missing or unclear information. | Pages 3, 4 | |
| Study risk of bias assessment | 11 | Specify the methods used to assess risk of bias in the included studies, including details of the tools used, how many reviewers assessed each study and whether they worked independently, and if applicable, details of automation tools used in the process. | Page 4 | |
| Effect measures | 12 | Specify for each outcome the effect measures e.g. risk ratio, mean difference used in the synthesis or presentation of results. | Page 4 | |
| Synthesis methods | 13a | Describe the processes used to decide which studies were eligible for each synthesis e.g. tabulating the study intervention characteristics and comparing against the planned groups for each synthesis item #5. | Page 3,4 | |
|  | 13b | Describe any methods required to prepare the data for presentation or synthesis, such as handling of missing summary statistics, or data conversions. | Page 3-5 | |
|  | 13c | Describe any methods used to tabulate or visually display results of individual studies and syntheses. | Page 5 | |
|  | 13d | Describe any methods used to synthesize results and provide a rationale for the choices. If meta-analysis was performed, describe the models, methods to identify the presence and extent of statistical heterogeneity, and software packages used. | Page 5 | |
|  | 13e | Describe any methods used to explore possible causes of heterogeneity among study results e.g. subgroup analysis, meta-regression. | Page 4 | |
|  | 13f | Describe any sensitivity analyses conducted to assess robustness of the synthesized results. | Page 6 | |
| Reporting bias assessment | 14 | Describe any methods used to assess risk of bias due to missing results in a synthesis arising from reporting biases. | Page 4 | |
| Certainty assessment | 15 | Describe any methods used to assess certainty or confidence in the body of evidence for an outcome. | Page 4 | |
| **RESULTS** | | |  |  |
| Study selection | 16a | Describe the results of the search and selection process, from the number of records identified in the search to the number of studies included in the review, ideally using a flow diagram. | Pages 5, 15 | |
|  | 16b | Cite studies that might appear to meet the inclusion criteria, but which were excluded, and explain why they were excluded. | Page 15 | |
| Study characteristics | 17 | Cite each included study and present its characteristics. | Page 5 | |
| Risk of bias in studies | 18 | Present assessments of risk of bias for each included study. | Page 3 | |
| Results of individual studies | 19 | For all outcomes, present, for each study: a summary statistics for each group where appropriate and b an effect estimate and its precision e.g. confidence/credible interval, ideally using structured tables or plots. | Pages 16, 17 | |
| Results of syntheses | 20a | For each synthesis, briefly summarise the characteristics and risk of bias among contributing studies. | Page 5 | |
|  | 20b | Present results of all statistical syntheses conducted. If meta-analysis was done, present for each the summary estimate and its precision e.g. confidence/credible interval and measures of statistical heterogeneity. If comparing groups, describe the direction of the effect. | Page 4-5 | |
|  | 20c | Present results of all investigations of possible causes of heterogeneity among study results. | Pages 5, 6, 7 | |
|  | 20d | Present results of all sensitivity analyses conducted to assess the robustness of the synthesized results. | Pages 7, 17 | |
| Reporting biases | 21 | Present assessments of risk of bias due to missing results arising from reporting biases for each synthesis assessed. |  | |
| Certainty of evidence | 22 | Present assessments of certainty or confidence in the body of evidence for each outcome assessed. | Pages 5, 6, 7, 16, 17 | |
| **DISCUSSION** | | |  |  |
| Discussion | 23a | Provide a general interpretation of the results in the context of other evidence. | Page 6 | |
|  | 23b | Discuss any limitations of the evidence included in the review. | Page 9 | |
|  | 23c | Discuss any limitations of the review processes used. | Page 9 | |
|  | 23d | Discuss implications of the results for practice, policy, and future research. | Page 9 | |
| **OTHER INFORMATION** | | |  |  |
| Registration and protocol | 24a | Provide registration information for the review, including register name and registration number, or state that the review was not registered. | Research Registry  reviewregistry1812 | |
|  | 24b | Indicate where the review protocol can be accessed, or state that a protocol was not prepared. | Page 1 | |
|  | 24c | Describe and explain any amendments to information provided at registration or in the protocol. |  | |
| Support | 25 | Describe sources of financial or non-financial support for the review, and the role of the funders or sponsors in the review. | Page 1 | |
| Competing interests | 26 | Declare any competing interests of review authors. | Page 1 | |
| Availability of data, code and other materials | 27 | Report which of the following are publicly available and where they can be found: template data collection forms; data extracted from included studies; data used for all analyses; analytic code; any other materials used in the review. | Page 1 | |

*From:*  Page MJ, McKenzie JE, Bossuyt PM, Boutron I, Hoffmann TC, Mulrow CD, et al. The PRISMA 2020 statement: an updated guideline for reporting systematic reviews. BMJ 2021;372:n71. doi: 10.1136/bmj.n71

For more information, visit: <http://www.prisma-statement.org/>

**Supporting Information Methods S2.** AMSTAR 2 Checklist


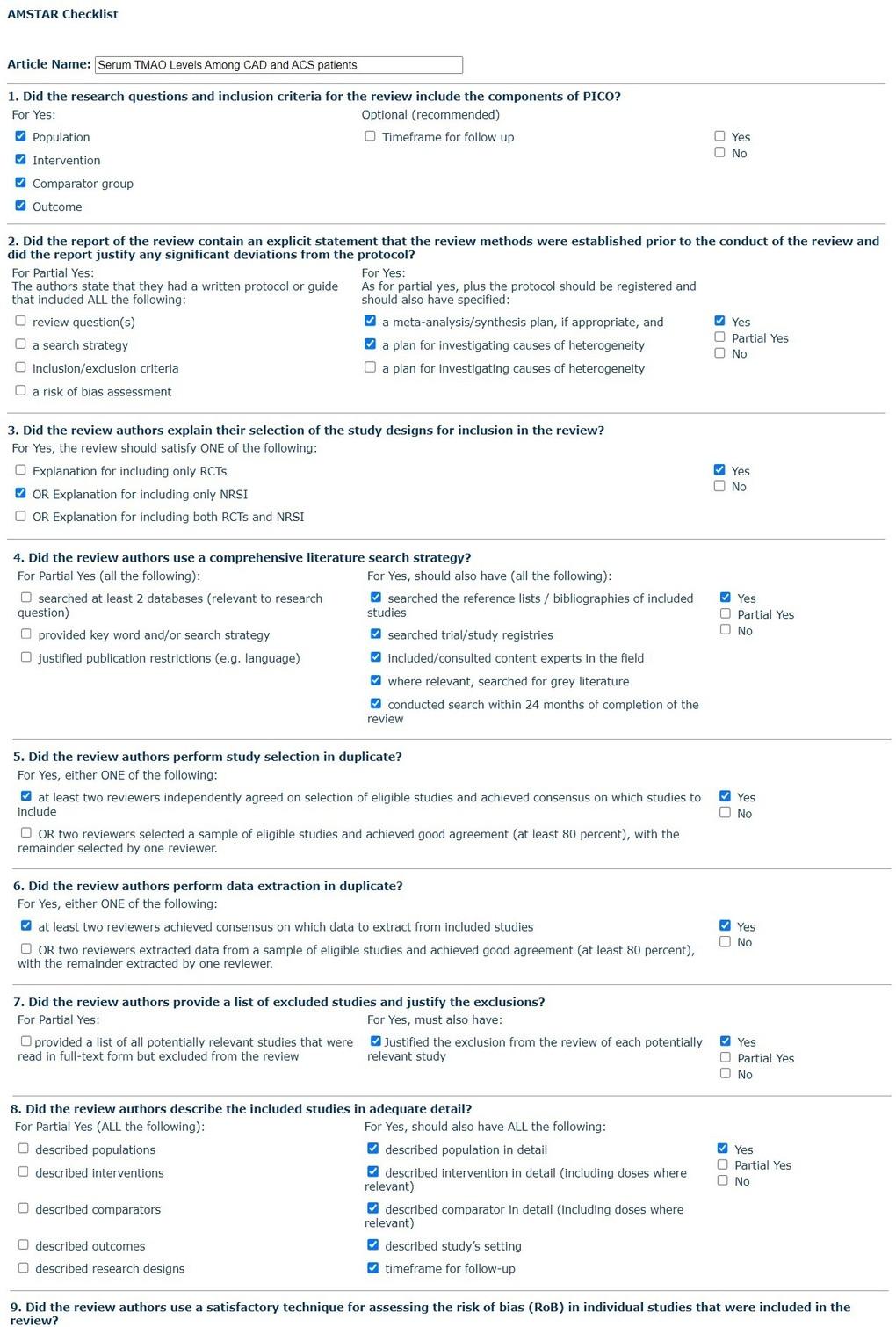


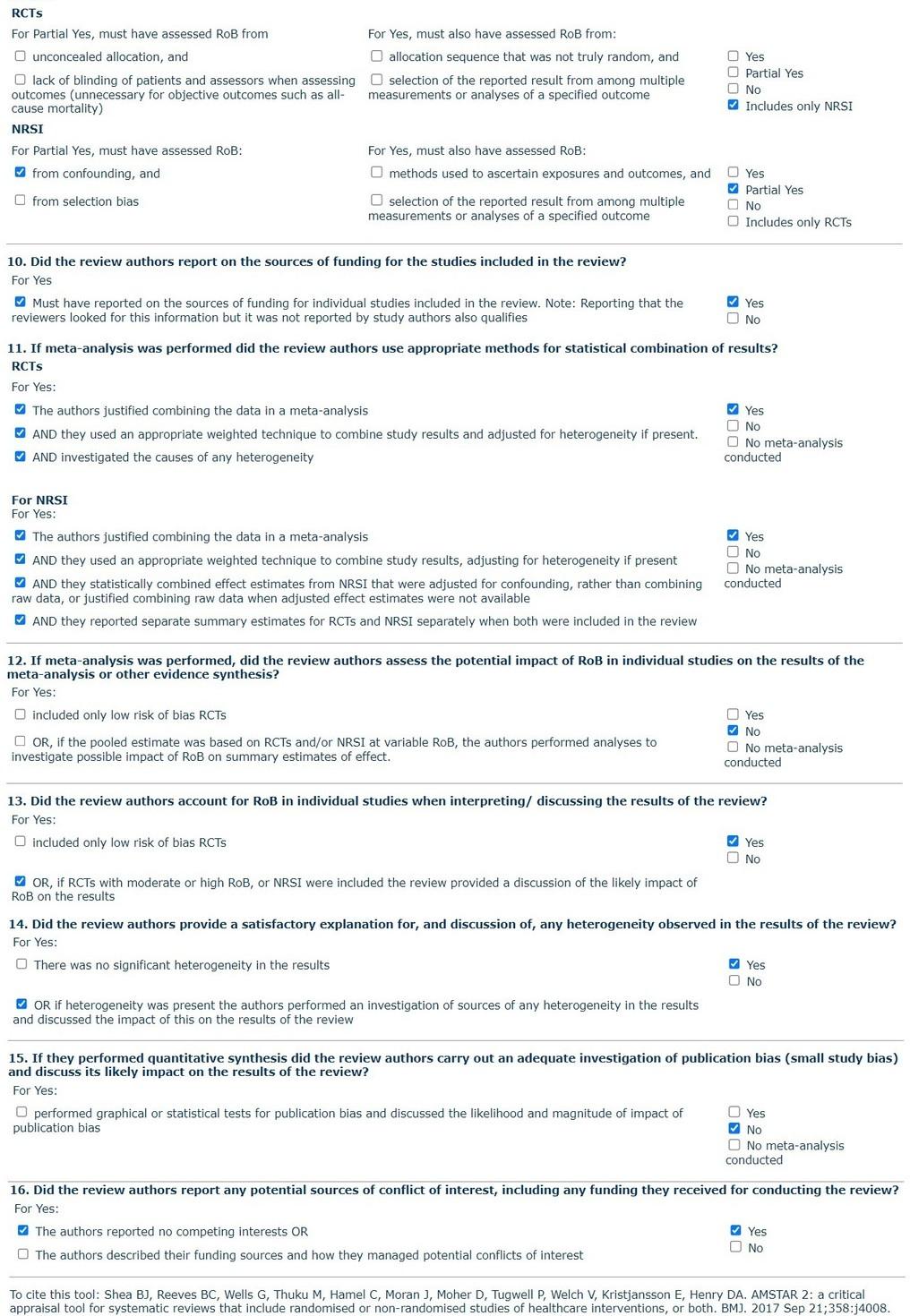


**Supporting Information Methods S3**. Details of the Search Strategy

| **Search Strategy** |
| --- |
| "Extracorporeal Membrane Oxygenation"[Mesh] OR "Extracorporeal Membrane Oxygenation" OR ECMO OR "Extracorporeal Life Support"[Mesh] OR "Extracorporeal Life Support" OR ECLS OR "extracorporeal membrane oxygenation" OR "extracorporeal life support"  AND  "Shock, Cardiogenic"[Mesh] OR "Cardiogenic Shock" OR "Cardiac Arrest"[Mesh] OR "Cardiac Arrest" OR "Cardiopulmonary Resuscitation"[Mesh] OR "Cardiopulmonary Resuscitation" OR "Fulminant"[Mesh] OR Fulminant  AND  "Myocarditis"[Mesh] OR "Myocarditis" OR "Myocarditis, Acute"[Mesh] OR "Acute Myocarditis" OR "Inflammatory Cardiomyopathy"[Mesh] OR "Inflammatory Cardiomyopathy" OR "Myocarditis, Viral"[Mesh] OR "Viral Myocarditis" OR "Myocarditis, Giant Cell"[Mesh] OR "Giant Cell Myocarditis" |

**Supporting Information Methods S4**. Addressing Heterogeneity

Between-study heterogeneity can be caused by one or more studies with extreme effect sizes that do not quite “fit in”. This may distort our pooled effect estimate, and it is a good idea to reinspect the pooled effect after such outliers have been removed from the analysis. We also want to attest if the pooled effect estimate we found is robust, meaning that it does not depend heavily on one single study. Therefore, we also want to know whether there are studies that heavily push the effect of our analysis to one direction. Such studies are called influential cases. Hence, we utilized outcomes excluding outliers detected by leave-one-out sensitivity for the primary and secondary endpoints that yielded any heterogeneity.

**Supplementary Figures 1:** Leave-one-out sensitivity analysis

Supplementary Figure 1A: Leave-one-out for cardiac Arrest


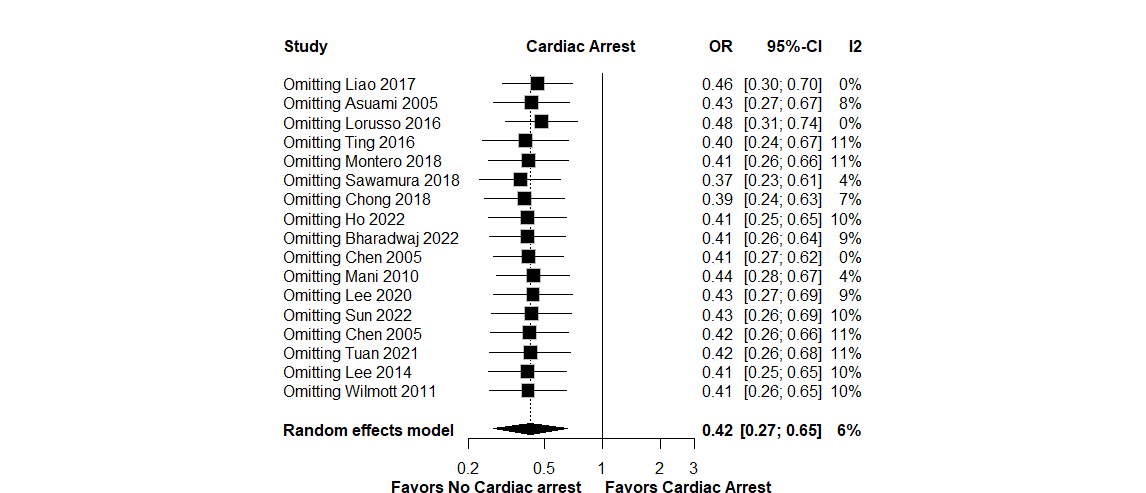


Supplementary Figure 1B: Leave-one-out for Mean Age
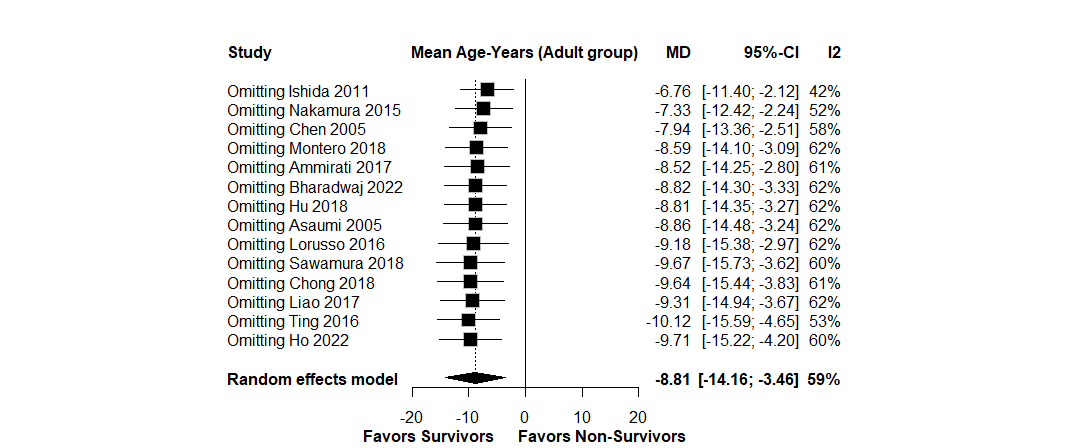


Supplementary Figure 1C: Leave-one-out for time from hospital admission to the initiation of ECMO.


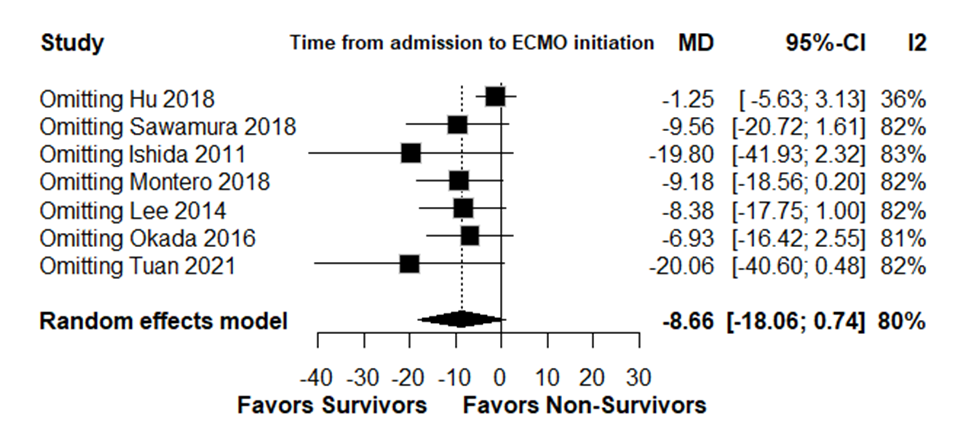


Supplementary Figure 1D: Leave-one-out for ECMO Duration


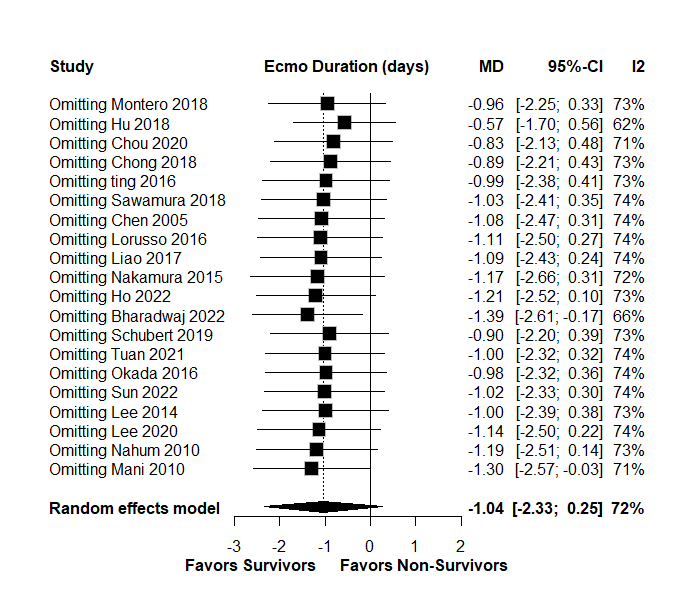


Supplementary Figure 1E: Leave-one-out for Mean serum Lactate levels


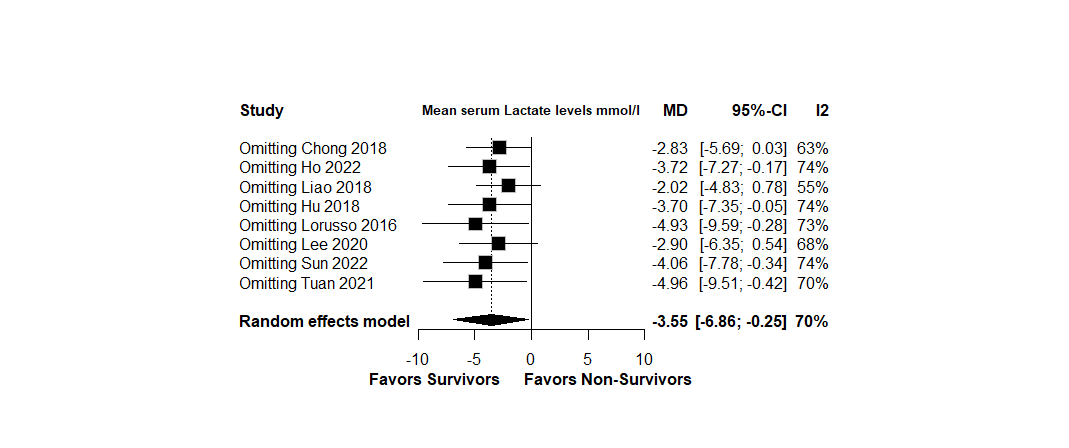


Supplementary Figure 1F: Leave-one-out for Pre-ECMO Left Ventricular Ejection Fraction (LVEF)


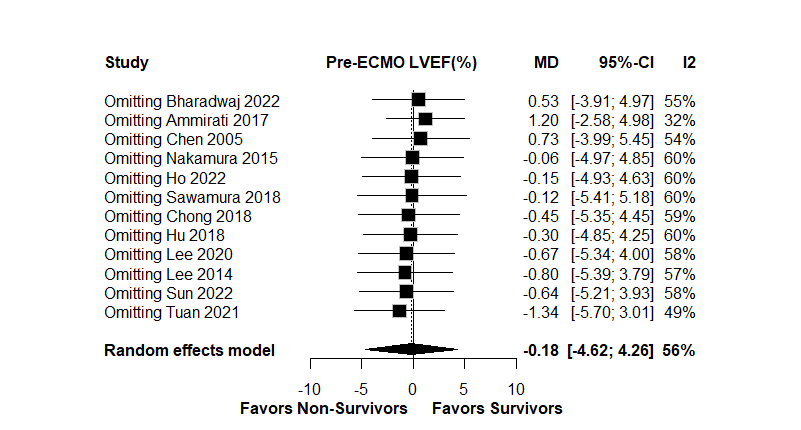


| **Supporting Information Methods S5**. Quality Assessment  For observational studies, the Newcastle–Ottawa scale (NOS) will be used to assess the quality of each study included. This scale comprises eight items within three domains: selection, comparability, and outcome/exposure. The NOS assigns a maximum of four points for selection, two for comparability, and three for outcome/exposure. A composite score was calculated to reflect the overall quality of each study, with scores > 7 indicating high quality.  Case series studies were assessed using the Joanna Briggs Institute (JBI) Critical Appraisal Tool.  When evaluating case series using the JBI critical appraisal tool, studies were judged as having a ‘low risk of bias’ if at least 8 items on the checklist scored ‘yes’; ‘some concerns of bias’ if 5–7 items scored ‘yes’; and a ‘high risk of bias’ if up to five items scored ‘yes’.  Publication bias in outcomes with 10 studies or more was assessed by funnel-plot analysis of point estimates according to study weights and asymmetry was tested by Egger’s regression test.  **Supplementary Table S1:** Quality Assessment for Cohort Studies | | | | | | | | | | |
| --- | --- | --- | --- | --- | --- | --- | --- | --- | --- | --- |
|  |  | **SELECTION** | | | | **COMPARABILITY** | **OUTCOME** | | | **TOTAL** |
| Author Year | | Representativeness | Selection of non-exposed cohort | Ascertainment of exposure | outcome of interest was not present at start of study | Comparability of 2 cohorts | Assessment of outcome | Follow  up | Adequacy of follow up | Total |
| Lee (2020) | | 1 | can't be determined | 1 | 1 | 0 | 1 | 0 | 0 | 4 |
| Lee (2014) | | 0 | 1 | 1 | 1 | 0 | 1 | 0 | 1 | 5 |
| Wilmott (2011) | | 0 | 1 | 1 | 1 | 2 | 1 | 1 | 1 | 8 |
| Wu (2007) | | 0 | 0 | 1 | 1 | 0 | 1 | 1 | 1 | 5 |
| Schubert (2019) | | 1 | 1 | 1 | 1 | 1 | 1 | 1 | 0 | 7 |
| Şık (2019) | | 0 | can't be determined | 1 | 1 | 1 | 1 | 1 | 1 | 6 |
| Teele(2011) | | 0 | 1 | 1 | 1 | 2 | 1 | 1 | 1 | 8 |
| Ho (2022) | | 0 | 0 | 1 | 1 | 0 | 1 | 0 | 0 | 3 |
| Liao (2017) | | 0 | 0 | 1 | 1 | 0 | 1 | 0 | 0 | 3 |
| Lin (2013) | | 0 | 0 | 1 | 1 | 0 | 1 | 0 | 0 | 3 |
| Lorusso (2016) | | 1 | 1 | 1 | 1 | 2 | 1 | 1 | 1 | 9 |
| Tuan(2021) | | 0 | 1 | 1 | 1 | 1 | 1 | 1 | 1 | 7 |
| Sun (2022) | | 0 | can't be determined | 1 | 1 | 2 | 1 | 1 | 1 | 7 |
| Chong(2018) | | 0 | 0 | 1 | 1 | 0 | 1 | 1 | 1 | 5 |
| Wu(2017) | | 0 | 1 | 1 | 1 | 0 | 1 | 1 | 1 | 6 |
| Lee (2021) | | 0 | 1 | 1 | 1 | 0 | 1 | 1 | 1 | 6 |
| Chen (2005) | | 1 | 1 | 1 | 1 | 0 | 1 | 0 | 0 | 5 |
| Asaumi(2005) | | 0 | 1 | 1 | 1 | 0 | 1 | 1 | 1 | 6 |
| Ammirati (2017) | | 0 | 1 | 1 | 1 | 2 | 1 | 1 | 1 | 8 |
| Li (2020) | | 0 | 0 | 1 | 1 | 0 | 1 | 0 | 0 | 3 |
| Matsumoto (2018) | | 1 | 0 | 1 | 1 | 0 | 1 | 1 | 1 | 6 |
| Mirabel (2011) | | 0 | 1 | 1 | 1 | 1 | 1 | 1 | 1 | 7 |
| Ting (2016) | | 0 | can't be determined | 1 | 1 | 0 | 1 | 0 | 0 | 3 |
| Zuo (2019) | | 0 | can't be determined | 1 | 1 | 0 | 1 | 1 | 1 | 5 |
| Weymann (2014) | | 0 | can't be determined | 1 | 1 | 0 | 1 | 1 | 1 | 5 |
| Bharadwaj (2022) | | 0 | can't be determined | 1 | 1 | 0 | 1 | 1 | 1 | 5 |
| Montero (2018) | | 0 | 1 | 0 | 1 | can't be determined | 0 | 1 | 1 | 4 |
| Nakamura (2015) | | 1 | 0 | 0 | 1 | 2 | 1 | 0 | 0 | 5 |
| Saito (2017) | | 0 | 1 | 1 | 1 | 2 | 1 | 0 | 0 | 6 |
| Sawamura (2018) | | 1 | 0 | 1 | 1 | 0 | 1 | 0 | 0 | 4 |
| Seguchi (2017) | | 0 | 1 | 1 | 1 | 2 | 1 | 1 | 1 | 8 |
| Chou(2020) | | 0 | 0 | 1 | 1 | 0 | 1 | 1 | 1 | 5 |
| Wang(2022) | | 0 | 1 | 1 | 1 | 0 | 1 | can't be determined | can't be determined | 4 |
| Ang (2008) | | 0 | 0 | 1 | 1 | 0 | 1 | 0 | 0 | 3 |
| Ishida (2011) | | 0 | 0 | 1 | 1 | 0 | 1 | 1 | 1 | 5 |
| Montero (2018) | | 0 | 1 | 0 | 1 | can't be determined | 0 | 1 | 1 | 4 |

**Supplementary Table S2:** Quality Assessment for Case Series

| Author (Year) | Were there clear criteria for inclusion? | Was the condition measured in a standard, reliable way for all participants included? | Were valid methods used for identification of the condition for all participants included in the case series? | Did the case series have consecutive inclusion of participants? | Did the case series have complete inclusion of participants? | Was there clear reporting of the demographics of the participants in the study? | Was there clear reporting of clinical information of the participants? | Were the outcomes or follow up results of cases clearly reported? | Was there clear reporting of the presenting site(s)/clinic(s) demographic information? | Was the statistical analysis appropriate? |
| --- | --- | --- | --- | --- | --- | --- | --- | --- | --- | --- |
| Duncan (2001) | Yes | Yes | Yes | Yes | Yes | No | Yes | Yes | Yes | Yes |
| Mani (2010) | Yes | Yes | Yes | Yes | Yes | No | Yes | Yes | Yes | Yes |
| Nahum (2010) | Yes | Yes | Yes | Yes | Yes | Yes | Yes | Yes | Yes | Yes |
| Okada (2016) | Yes | Yes | Yes | No | No | Yes | Yes | Yes | Yes | Yes |
| Hu (2018) | Yes | Yes | Yes | Yes | Yes | Yes | Yes | Yes | Yes | Yes |
| Jung (2016) | Yes | Yes | Yes | Yes | Yes | Yes | Yes | Yes | No | Yes |

**Figures 2.** Publication Bias Assessment

Supplementary Figure 2A: Funnel plot for Cardiac Arrest


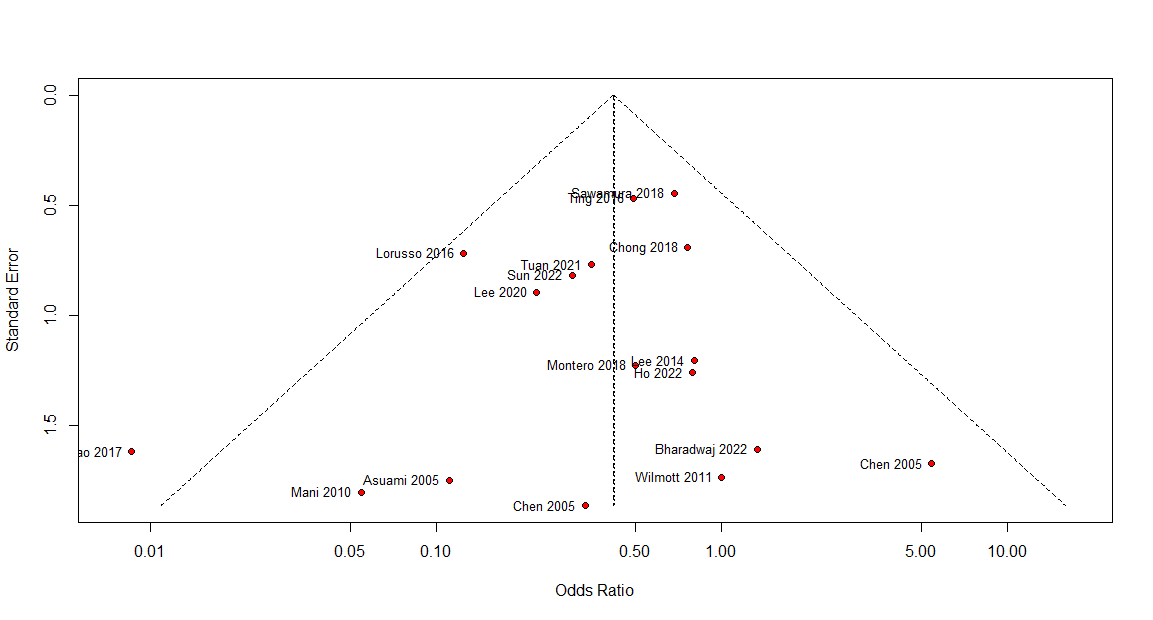


Supplementary Figure 2B: Funnel plot for Mean Age


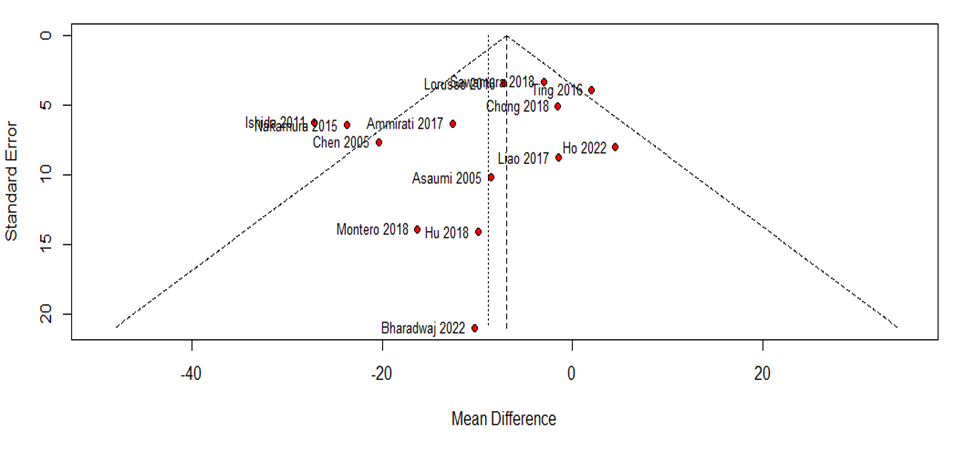


Supplementary Figure 2C: Funnel plot for ECMO Duration


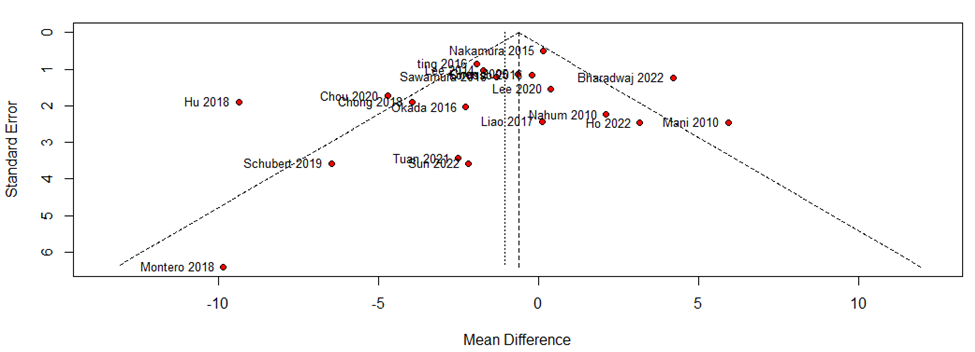


Supplementary Figure 2D: Funnel plot for Pre-ECMO Left Ventricular Ejection Fraction (LVEF)


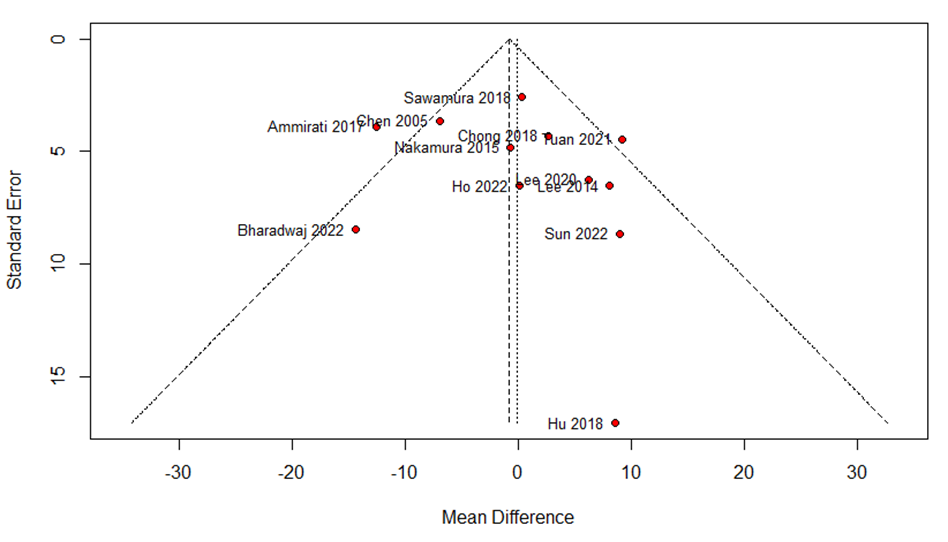


**Supplementary Table S3.** Egger’s regression test

*Supplementary Table S3A: Egger’s regression Test for cardiac arrest*

| **Egger’s Regression Test for cardiac arrest** | | | |
| --- | --- | --- | --- |
| **Intercept** | **95% CI** | **t** | **p-value** |
| 0.165 | (-0.46; - 0.79) | 0.518 | 0.6114978 |

*Supplementary Table S3B: Egger’s regression Test for Mean age*

| **Egger’s Regression Test for Mean age** | | | |
| --- | --- | --- | --- |
| **Intercept** | **95% CI** | **t** | **p-value** |
| -1.145 | (-2.95 - 0.66) | -1.243 | 0.2376068 |

*Supplementary Table S3C: Egger’s regression Test for* ECMO Duration

| **Egger’s Regression Test for** **ECMO Duration** | | | |
| --- | --- | --- | --- |
| **Intercept** | **95% CI** | **t** | **p-value** |
| -0.817 | (-2.39; - 0.76) | -1.017 | 0.3226534 |

*Supplementary Table S3D: Egger’s regression Test for* Pre-ECMO Left Ventricular Ejection Fraction (LVEF)

| **Egger’s Regression Test for LVEF** | | | |
| --- | --- | --- | --- |
| **Intercept** | **95% CI** | **t** | **p-value** |
| 0.771 | (-1.44; - 2.99) | 0.682 | 0.5107301 |
